# Community assembly, functional traits, and phylogeny in Himalayan river birds

**DOI:** 10.1002/ece3.9012

**Published:** 2022-06-17

**Authors:** Ankita Sinha, Nilanjan Chatterjee, Ramesh Krishnamurthy, Steve J. Ormerod

**Affiliations:** ^1^ Wildlife Institute of India Dehradun India; ^2^ Faculty of Forestry University of British Columbia Vancouver British Columbia Canada; ^3^ Water Research Institute, Cardiff School of Biosciences Cardiff University Cardiff UK; ^4^ Freshwater Biological Association Ambleside, Cumbria UK

**Keywords:** assembly processes, environmental filtering, functional traits, phylogeny, river bird, RLQ

## Abstract

Heterogeneity in riverine habitats acts as a template for species evolution that influences river communities at different spatio‐temporal scales. Although birds are conspicuous elements of these communities, the roles of phylogeny, functional traits, and habitat character in their niche use or species' assembly have seldom been investigated. We explored these themes by surveying multiple headwaters over 3000 m of elevation in the Himalayan Mountains of India where the specialist birds of montane rivers reach their greatest diversity on Earth. After ordinating community composition, species traits, and habitat character, we investigated whether river bird traits varied with elevation in ways that were constrained or independent of phylogeny, hypothesizing that trait patterns reflect environmental filtering. Community composition and trait representation varied strongly with increasing elevation and river naturalness as species that foraged in the river/riparian ecotone gave way to small insectivores with direct trophic dependence on the river or its immediate channel. These trends were influenced strongly by phylogeny as communities became more clustered by functional traits at a higher elevation. Phylogenetic signals varied among traits, however, and were reflected in body mass, bill size, and tarsus length more than in body size, tail length, and breeding strategy. These variations imply that community assembly in high‐altitude river birds reflects a blend of phylogenetic constraint and habitat filtering coupled with some proximate niche‐based moulding of trait character. We suggest that the regional co‐existence of river birds in the Himalaya is facilitated by this same array of factors that together reflect the highly heterogeneous template of river habitats provided by these mountain headwaters.

## Graphical abstract

We explored the potential role of environmental gradients, species traits and phylogenetic relatedness in shaping assemblages of specialist river birds in the Himalayan mountains ‐ where this group reaches its greatest richness on Earth. Assemblage composition and trait representation changed markedly with elevation through a combination of habitat filtering and functional clustering reflecting large habitat heterogeneity, but phylogenetic patterns were also clear.

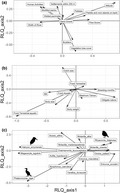

## 1 INTRODUCTION

Understanding how species assemble into communities is one of the most fundamental themes in ecology (Gerhold et al., 2015; Götzenberger et al., 2012; Weiher et al., 2011). Key postulates are that evolutionary forces, environmental conditions, and inter‐species interactions combine to structure local communities (Cavender‐Bares et al., 2009; Webb et al., 2002), with prominent theories proposing either neutral or deterministic processes (Chase & Myers, 2011; Kembel, 2009; Swenson & Enquist, 2009). Neutral theory holds that species initially have quasi‐identical requirements, and communities become structured by some dynamic balance between species loss through extinction, immigration and speciation through genetic drift (Kimura, 1991). Conversely, niche‐based concepts emphasize how environmental factors determine assembly through filtering mechanisms that limit the occurrence of species with similar traits (Kraft et al., 2015). While niche overlap—also known as limiting similarity—is expected to exclude similar species from co‐existing (Macarthur & Levin, 1967), environmental filtering and niche shifts act to moderate the extent to which similar species co‐occur in similar habitat conditions (Gerhold et al., 2015; Ulrich et al., 2018; Weiher et al., 2011).

An important proviso in studying species and trait assembly in communities is that species relatedness should be controlled or represented in order to eliminate phylogeny as a potential confound (Cadotte & Tucker, 2017; Kraft et al., 2015; Mayfield & Levine, 2010). Phylogenetic analyses can account for trait expression at the species level, thus enabling insights into the evolution of habitat preferences, species function, and distribution patterns (McGill et al., 2006; Webb et al., 2002). Typical analyses attempt to understand whether ecologically relevant traits are conserved or modified along any given phylogeny, thereby providing evidence about the roles of environmental filtering and competitive segregation in assembly processes (Cavender‐Bares et al., 2009; Pavoine et al., 2008; He et al., 2018; reviewed in Cadotte et al., 2019). Ideally, investigations aimed at understanding communities should blend field observations with some assessment of the functional and phylogenetic identities of the component species (McGill et al., 2006; Winemiller et al., 2015; Xu et al., 2017).

Given that ecosystem character changes in time and space, conditions under which species assemble and co‐exist must also vary (McGill et al., 2006). Such environmental gradients offer a means to test competing assembly theories, for example, by revealing relationships between environmental conditions and the morphological, physiological, or behavioral traits of the species involved (Cavender‐Bares et al., 2004; Dehling et al., 2014). Traits also reflect species' roles or functions within communities and can reveal mechanisms that affect distributional patterns along habitat gradients (Kraft et al., 2007). In terrestrial ecosystems, for example, competition, trait expression, and environmental filtering along elevational gradients can have marked effects on bird communities (Chiu et al., 2020; Dehling et al., 2014; Ding et al., 2019; He et al., 2018; Machac et al., 2011; McCain, 2009; Ulrich et al., 2018).

Among all ecosystems, rivers have received considerable emphasis in community ecology (Altermatt et al., 2020; Robinson et al., 2002; Ward et al., 1998), including seminal assessments of assembly rules, environmental filtering, and trait‐based studies (Heino et al., 2015; Poff, 1997). In part, this interest reflects the pronounced environmental gradients represented by rivers both longitudinally and among contrasting river basins that together have created a diverse habitat template into which species have proliferated (Terui et al., 2021; Townsend & Hildrew, 1994). Growing concern about the global status of freshwater ecosystems is also prompting interest in interactions between natural biodiversity patterns in rivers and the effects of environmental change (see Dudgeon et al., 2006).

So far, little of the research effort into community assembly has focused on high‐energy river systems in mountain landscapes, where large altitudinal ranges, complex topography, and geomorphological dynamism give rise to pronounced ecological gradients with large species turnover (Jacobsen et al., 1997; Ormerod et al., 1994). Moreover, despite being conspicuous components of the global riverine fauna, river birds have been neglected in fundamental studies of mechanisms structuring communities, especially in mountainous areas (Manel et al., 2000; Sinha et al., 2019). One such region, the Himalayan Mountains, has the Earth's most diverse communities of specialist birds of montane rivers (Buckton & Ormerod, 2002)—thus prompting questions about evolutionary mechanisms that have allowed their coexistence. We focused on this group specifically because (i) they utilize production from the river channel either by direct aquatic foraging or by exploiting the subsidy of food resources that reaches the riparian zone by export from the river channel (Buckton & Ormerod, 2008; Petersen et al., 2004); (ii) they occupy habitats created specifically by the fluvial geomorphological system linked to high energy rivers for most of their life cycle—unlike birds in riparian forest or other freshwater ecosystems (Buckton & Ormerod, 2002); and (iii) these circumstances allow for a relatively straightforward quantification of relationships between specific river features and bird distribution, and hence hypothesis testing. Marked diversity and distinctness in habitat use among this group of birds has led already to some speculation about the roles of environmental filtering and niche partitioning, but there has been no formal analysis using current methods, and no attempts to assess phylogenetic effects in community assembly (Buckton & Ormerod, 2008).

In this study, we use specialist river birds to examine Southwood's original premise (Southwood, 1977; Southwood, 1988), restated for rivers by Townsend and Hildrew (1994), that habitats provide the templet through which evolutionary forces act with phylogeny to determine species' life history. In turn, the resulting contrasts in species' traits act to determine how communities can contain multiple species while also influencing how communities change along environmental gradients. Specifically, we investigated river birds along multiple headstreams in the northwest Himalayan mountains of India, hypothesizing that river bird communities reflect detectable trait–environment relationships arising from environmental filtering. We asked: (1) are there non‐random patterns in species distribution and species' traits that reflect trait–environment relationships?; and (2) are local species pools a result of common phylogenetic ancestry or convergence in response to environmental or biotic filters acting on regional communities? Addressing the first of these questions allowed us to quantify community change largely in relation to elevation while the second helped to identify how trait expression along this elevation gradient reflected filtering beyond the constraints of phylogeny.

## 2 MATERIALS AND METHODS

### 2.1 Study area

This particular study focused on river‐dependent birds along snow‐fed and perennial headstreams in the western Himalaya of India, specifically the Bhagirathi and Amrutganga rivers in Uttarakhand, and the Tirthan River in Himachal Pradesh (Figure S1). Sites were selected over an altitudinal gradient from 330 to 3100 m and represented a range of habitat types from near‐natural environments in protected areas to river stretches affected by human activities such as farming or urbanization at lower altitude. Climatological conditions vary from sub‐tropical to temperate (Mathur & Naithani, 1999; Sinha, 2021), with drainage varying from glacial meltwater, rainwater, and underground springs. Areas above 1500 m in the northwest Himalaya are highly seasonal with cold winters and mild summers (Barve, 2017).

Of the specific study locations, the Tirthan River (N 31.6396° E 77.401°) is a major tributary of the River Beas in the Indus system. Here, survey locations encompassed river reaches between 1400 and 2300 m in the Great Himalayan National Park Conservation Area in the Kullu district where the natural terrain is characterized by numerous high ridges, deep gorges, and narrow channels (Mathur & Naithani, 1999). The buffer zone of the protected area has hamlets with orchards and other small‐scale agricultural practices and game fishing for introduced Brown Trout (*Salmo trutta*). Inside the protected area, the river flow is uninterrupted while the riparian zone is relatively pristine with conifers and broadleaf woodland. In the Gangetic headwaters, river reaches were surveyed along the Bhagirathi (N 30.7564° E 78.5781°) and its associated low‐order tributaries covering an elevation gradient between 300 and 3300 m. Riparian vegetation consists of conifers at higher elevations and subtropical vegetation at the foothills. In this non‐protected area within the administrative districts of Uttarkashi, Tehri, and Dehradun, habitats have been modified by a range of anthropogenic pressures from agriculture and settlements (Sinha et al., 2019). Sites in the Amrutganga valley (N 30.466° E079.269°) are part of the Kedarnath Wildlife Division in Chamoli district where riparian land‐use ranges from well‐vegetated river reaches to small villages with traditional agriculture (Barve, 2017) between elevations of 1400 and 2650 m.

### 2.2 Bird surveys

Bird species that depend on aquatic production and occupy the riparian zone of Himalayan rivers were known from reconnaissance surveys and previous studies (Buckton & Ormerod, 2002; Manel et al., 2000). For the current analysis, replicate surveys of the 68 reaches were undertaken in 2017–2018 in the pre‐monsoon period (March–June), thereby capturing the breeding season of almost all the target birds. The banks were walked by the same observer (AS) during early morning (06.00 to ±10.00) and late afternoon (15.00 to ±18.00) following a previously established field design in which three visits were made to each river segment of 500 m length on different days (Buckton, 1998). This visit frequency is considered appropriate for detecting specialist river birds that occupy linear territories (D'Amico & Hemery, 2003). The order of visits to each site within the basin were randomized as far as possible while ensuring minimum distances of 30–50 km between the sampling sites on consecutive days to maintain spatial independence (McCarthy et al., 2013). Species were recorded as present if they were observed during any of the three visits, while numbers of individuals of each species were recorded on every visit and eventually converted to mean numbers per visit. Bird species occurring in less than five river reaches were excluded from further analysis.

### 2.3 Trait information

Data on species traits were obtained from existing literature and field surveys (2014–2019; Sinha, 2021). Elevational distribution patterns were identified from surveys in both breeding and wintering seasons (Sinha, 2021). Morphometric measurements such as body size, average body mass, bill length, wing length, tail length, and tarsus length, along with clutch size and diet, were gleaned from available literature (Ali & Ripley, 1968; Buckton & Ormerod, 2008). Wing length was strongly inter‐correlated (*r* > .7) with other measures of body size and so was dropped from subsequent analysis to reduce multicolinearity, while traits that summarized foraging and breeding behavior were retained in assessments of trait–environment relationships.

### 2.4 River habitat characterization

Physical variables describing the river and riparian zone at each site were recorded alongside bird surveys to capture information on river channel structure, flow character, bank structure, riparian vegetation, and adjacent land use following methods developed by Sinha et al. (2019) after Raven et al. (1998). Observations were made at two different scales, respectively: (i) perpendicular transects or “spot checks” at 10 points every 50 m along each 500 m reach, specifically recording progressive lateral changes at each point in flow character and habitat features from the channel to the riparian zone; and (ii) “sweep up” assessments that recorded features over the whole 500 m survey site. The resulting data blended quantitative and semi‐quantitative methods, for example, with features recorded as present (<33% of the survey reach) or extensive (>33%), or on a 6‐point scale (rare: 1–20% cover; occasional 21–40%; frequent 41–60%; abundant 61–80%; and dominant 81–100%). A more extensive description of the variables recorded and their ability to detect variations among locations is provided by Manel et al. (2000).

### 2.5 Statistical analysis

In outline, our statistical analysis involved (i) ordination of species, trait, and habitat variations along the large elevational range of our sites; (ii) assessment of trends in taxonomic, phylogenetic, and functional diversity; and (iii) analysis of phylogenetic relatedness among the river species recorded to enable an unconfounded assessment of trait variations as community composition changed with environmental character.

### 2.6 Community trends: RLQ analysis

Trait and community responses to environmental gradients were ordinated using an RLQ framework, a multivariate technique that summarizes joint structure among matrices (Dray et al., 2014; Dray & Legendre, 2008). In our case, these three matrix Tables L (species distribution across river reaches surveyed as 15 species abundances*68 sites), R (environmental characteristics of samples: 68 sites*12 environmental variables), and Q (species traits: 15 species*8 traits) were analyzed separately using different ordination methods in “ade4” package in R (Dray & Dufour, 2007). The L‐species table was analyzed using correspondence analysis (CA), while the R‐environmental variables table and Q‐trait table were analyzed by a Hill–Smith PCA combining quantitative and qualitative variables using CA species scores as a column weight to couple Q and L (Brown et al., 2014). In trait analysis, the RLQ approach crosses traits and environmental variables weighted by species abundances with significant effects tested using a two‐step permutation procedure (25,000 permutations). The Dray et al. (2014) Model 2 permutes the rows of the L matrix to test the null hypothesis that no relationship exists between species abundance data with fixed traits and their environment; the alternate hypothesis being that the environment influences the distribution of species with fixed traits. Model 4 permutes the columns of dataset L to test the null hypothesis that species composition is not influenced by species traits given fixed environmental characteristics; the alternate hypothesis being traits influence the composition of assemblages (Dray et al., 2014).

### 2.7 Phylogenetic and functional trees

As a basis for all subsequent phylogenetic analysis, we prepared a phylogenetic tree for the species in our community by trimming from the original phylogeny available from www.birdtree.org (Jetz et al., 2012; Figure S2). We used the R packages “ape” and “phytools” (Paradis et al., 2004; Revell, 2012) to obtain a consensus tree for our 15 target species using a pseudo‐posterior distribution (https://birdtree.org/subsets/) from 1000 random samples from the “backbone tree” after applying the 50% majority rule (i.e., the proportion of a split to be present in all trees) prior to modeling inter‐specific variation across the phylogeny.

Bird species were classified into a functional tree using the quantitative morphological traits and qualitative feeding traits collected from Ali and Ripley (1968). We used “gower” distance to calculate the pairwise distances between species while the UPGMA clustering method was used to convert the species‐wise trait distances into branches in which the species formed the tips labeled with the aid of the “phangorn” package (Schliep, 2011).

### 2.8 Diversity metrics

While species richness (SR) offered a simple measure of taxonomic diversity at each site, phylogenetic diversity (PD) was calculated as Faith's PD index, or the sum of all branch lengths of the phylogeny connecting all species at a site (Faith, 1992), using the function “pd” in R package *picante* (Kembel et al., 2010). The branch lengths are taken to represent evolutionary time, with higher PD indicating group of species that are more evolutionarily apart in time (Tucker et al., 2019). Absolute functional diversity was estimated as functional richness, FRic (Villéger et al., 2008), which represents the multidimensional volume of functional space occupied by the species within a community (Villéger et al., 2008). We estimated FRic by computing the pairwise distance between all birds (branch lengths of the functional dendrogram for species within a community) at every site which had more than two species. The dimensions of the functional distance matrix were condensed using PCoA to estimate the convex hull volume of functional spaces for species within a community in R using the package “*FD”* (Laliberté et al., 2014).

Dispersion metrics for continuous traits were measured by quantifying the community‐weighted mean (CWM) of traits showing phylogenetic signal using the “dbFD” function in the “FD” R package (Laliberté et al., 2014). The rationale behind this was that environmental filtering would lead to a decrease in trait range or variance in communities (e.g., Graham et al., 2009; Zhang et al., 2020). CWM statistics were calculated by taking averages of trait values of species that were present in each site (communities), weighted by species abundance.

Mean pairwise phylogenetic distance (MPD) and mean pairwise functional distance (MFD) at each site (Webb et al., 2002) were derived from the average functional or phylogenetic distance between each pair of species that co‐existed at each site calculated as:

$$\mathrm{MPD}\left(\text{or}\;\mathrm{MFD}\right)=\frac{\sum_{i}^{n}\sum_{j}^{n}\delta_{i,j}}{n}\left(i\neq j\right)$$

where *n* is SR in each band, and $\delta_{i,j}$ is the pairwise functional or phylogenetic distance (Euclidean distance) between species *i* and species *j*.

For a phylogenetic or functional tree “T” with a set of species “*n*” represented by a subset of branching nodes, the MPD of “*n*” is equal to the average of the distances of all possible simple paths in “T” that connect pairs of nodes in “*n*” (Tsirogiannis & Sandel, 2014). We compared these indices to 1000 randomized communities to test whether the functional and phylogenetic community structures differed from random expectations. For this, we used the function “*ses. Mpd*” in package *picante* (Kembel et al., 2010) to generate random communities by shuffling the tips of the branches of the phylogenetic and functional trees used to calculate distance matrices for the entire community of river birds keeping the SR constant. This procedure assumes that all species could colonize habitats across the whole gradient but are excluded due to local biotic and abiotic factors. We calculated the standardized effect size (SES) of MPD and MFD for each site comparing the observed values versus the expected values from the null communities (Kembel et al., 2010). The SES aids in inferring community assembly processes like environmental filtering and competition. When traits and lineages are conserved (i.e., with phylogenetic signals) with SES values <0, it indicates that communities are phylogenetically and functionally clustered and are shaped by environmental filtering. Community overdispersion with SES >0 for MPD and MFD values is taken to indicate competitive exclusion (Webb et al., 2002).

### 2.9 Phylogenetic signal

Among the available indices available to characterize phylogenetic signals in trait data, Blomberg's *K* is the most widespread and is considered to capture the effect of trait evolution (Blomberg et al., 2003; Münkemüller et al., 2012). This is based on an approach in which the magnitude of independent contrasts has smaller variance if related species are similar to each other in trait character. Observed versus expected contrast variances were compared under a null model created by swapping the tips of the phylogenetic tree to test for significance differences (Blomberg et al., 2003). When *K* approaches 1, trait evolution follows a mode of evolution that is consistent with Brownian motion (i.e., random walk), whereas for *K* > 1 and <1, respectively, close relatives are more similar or less similar than expected, indicating a strong phylogenetic signal (Blomberg et al., 2003). Using all the traits measured as continuous variables (body mass, body size, bill length, tarsus length, and clutch size), we calculated Blomberg's *K* and K* as reported by Münkemüller et al. (2012) using the R package “phylosignal” (Keck et al., 2016). While K* is calculated with mean of raw trait values, *K* is the phylogenetically corrected mean. However, both of these measures are reported to have high correlation (Blomberg et al., 2003). The significance of *K* (*p*‐value) was calculated by comparison to a null distribution (Yang et al., 2014). We also used Moran's correlograms, plotted using the function “phyloCorrelogram” from the package “phylosignal” (Keck et al., 2016), to assess how phylogenetic autocorrelation changed across different phylogenetic distances. Originally a measure of spatial autocorrelation, when used in phylogenetic analysis, Moran's I assesses phylogenetic proximity among species to describe the relationship between cross‐taxonomic trait variation and phylogeny.

All statistical tests were performed with R software version 3.6.2 (R Core Team, 2019).

## 3 RESULTS

### 3.1 General species composition

Field surveys recorded 483 individual birds belonging to 15 species from the families Alcedinidae, Motacillidae, Muscicapidae, Cinclidae, Ibidorynchidae, and Charadriidae. This included two river chats, three wagtails, two forktails, a thrush, and a dipper which are all widespread in the Western Himalaya (Figure S3). The Plumbeous Water Redstart (*Rhyacornis fuliginosa*) was the most abundant bird recorded in all the three river basins while Ibisbills (*Ibidorhyncha struthersii*) were recorded from just two river reaches in the Bhagirathi basin (Figure S3). All 15 species were recorded from the Bhagirathi basins, while 8 species were recorded in each of Amrut Ganga and Tirthan River basins. Sites with most species were from the Bhagirathi basin at elevations between 1000 and 1500 m (Figure S4).

### 3.2 Community trends from RLQ analysis

**FIGURE 1:**
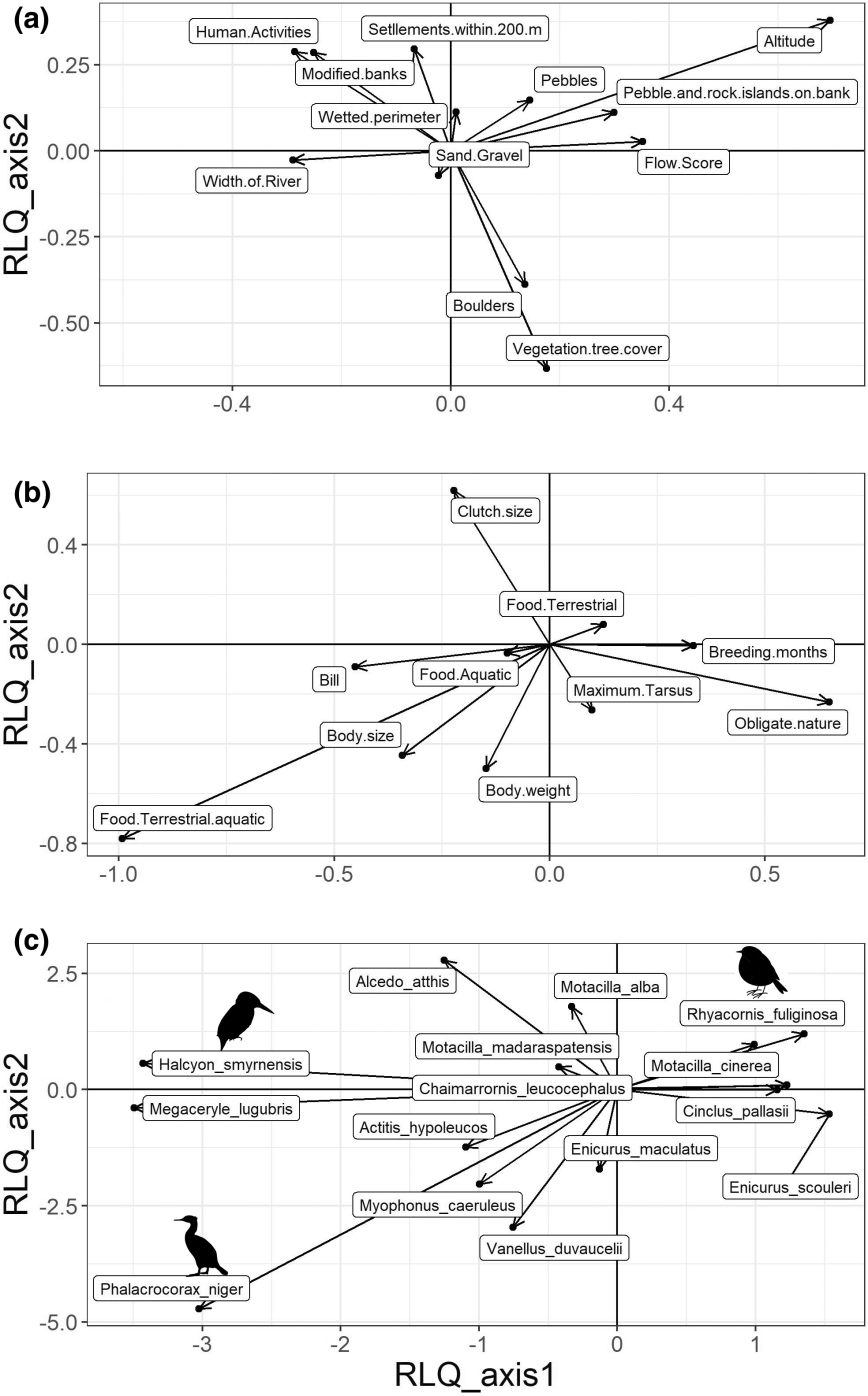
Biplot depicting the first two axes of the RLQ multivariate analysis. Axes and scale are same for figures of all plots which represent projections in the plane of the first two main components of (a) environmental variables, (b) species traits, and (c) bird species

RLQ analysis illustrated how traits, species, and habitat features varied together. Two axes explained 90.3% of the total inertia in the three tables, also accounting for most variability (>72–79%) along the first two axes of the environmental variables (R‐table) and species' functional traits (Q‐table) separately (Table S1). Traits and environmental variables were particularly strongly related to the first RLQ axis (Figure 1a,b).

Both major RLQ axes were related strongly to elevation. The first axis reflected a significant altitudinal trend toward narrower river stretches with faster flows, well‐vegetated banks, and channels with boulders and pebbles, while sandy banks, altered riparian cover, human settlements, and human activities declined (Figure 1a). Feeding traits correlated significantly with this axis as species using more terrestrial prey from the river margins increased toward higher elevations (e.g., Plumbeous Water Redstart and White‐capped Water Redstart (*Phoenicurus leucocephalus*)), whereas species using a blend of terrestrial and aquatic prey declined (e.g., White‐throated Kingfisher (*Halcyon smyrnensis*) (Figure 1b). Simultaneously, species using aquatic prey solely such as the Brown Dipper (*Cinclus pallasii*) and Little Forktail (*Enicurus scouleri*) increased along this axis. Overall, the contribution of “aquatic” feeding was minimal and neutral as aquatic‐feeding species persisted at both ends of the axis.

The second axis of the RLQ mostly represented a significant decline in riparian vegetation cover and boulder‐strewn banks but an increase in pebble banks and islands at a higher elevation—typical of upland braided reaches. Bird traits varying significantly on this axis included an increase in clutch size, but a decline in body size, tarsus size, bill size, and aquatic/terrestrial foraging as species such as River Lapwing (*Vanellus duvaucelii*), Common Sandpiper (*Actitis hypoleucos*), Blue Whistling thrush (*Myophonus caeruleus*), and Spotted Forktail (*Enicurus maculatus*) dropped out of the community (Figure 1).

Strong, significant relationships among the trait, habitat, and species abundance data were corroborated by the global RLQ permutation test (*p* < .001 for Model 2 of Dray et al., 2014). This held across all regions suggesting a uniform pattern in the species–trait–environment relationship at the community level. Model 2 was rejected (*p* = .0005) and Model 4 was accepted (*p* = .608) together suggesting that the environment plays a key role in shaping assemblage patterns such that (i) species distributions were influenced by environmental conditions, dominantly through changes related to elevation; and (ii) species composition reflected significant variations in trait character that also tracked elevation on both major axes.

### 3.3 Diversity gradients

**FIGURE 2:**
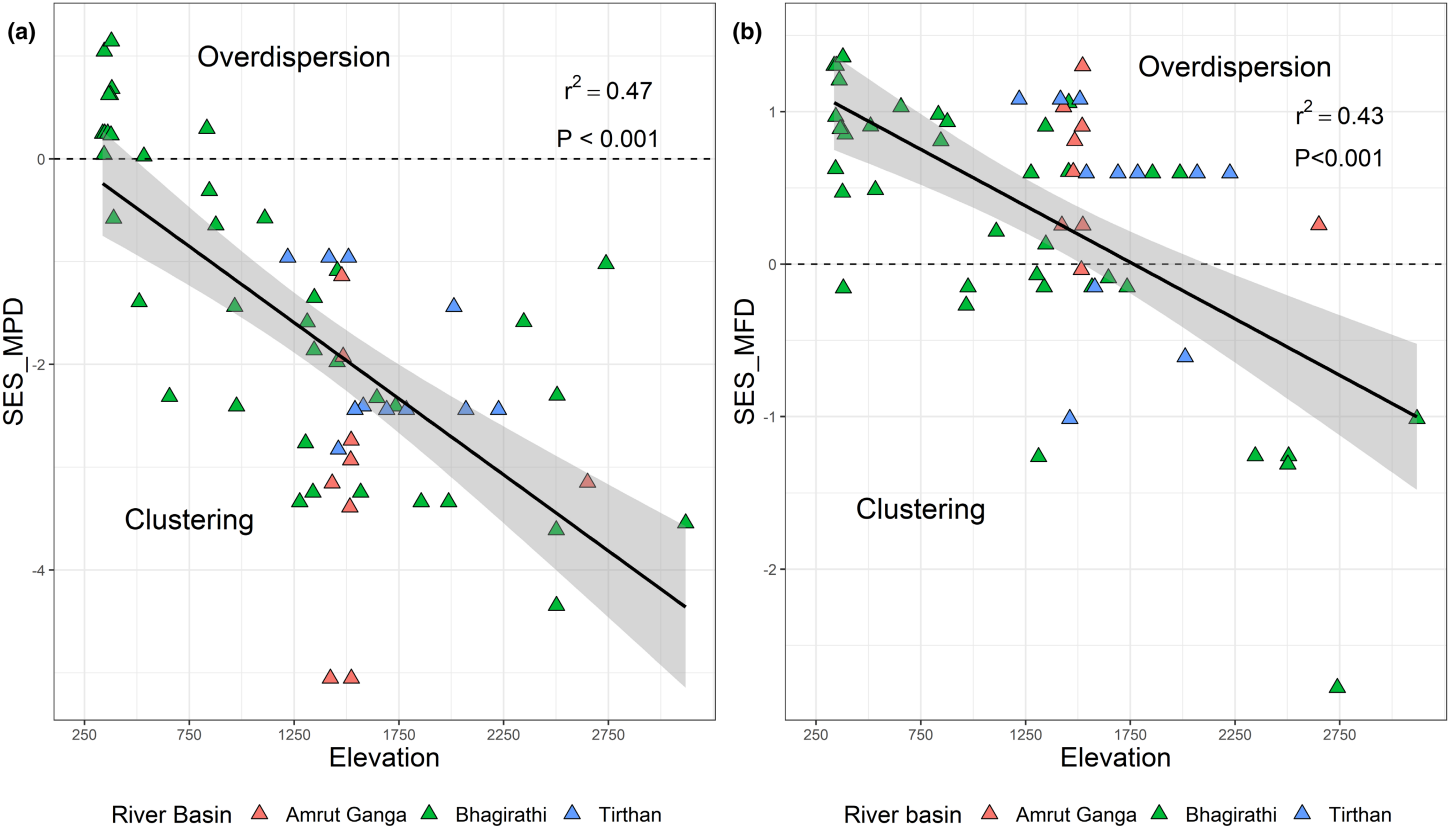
Plots showing elevational trends of standard effect size of mean phylogenetic distance (SES_MPD; panel (a)) and standard effect size of mean functional distance (SES_MFD; panel (b)) of breeding river birds across the 68 river reaches in the western Himalaya

None of the three diversity indices (SR, phylogenetic diversity (Faith's PD), and functional diversity (FRic)) was related clearly to elevation and, in all cases, there was substantial variation across sites (Figure S4). After controlling for SR, however, elevation affected phylogenetic composition such that communities at higher elevations (>1000 m) consisted of species that were more closely related than expected by chance (Figure 2)—i.e., smaller‐bodied river chats, forktails, wagtails, and Brown Dipper. SES_MFD and SES_MPD also indicated that communities were functionally more clustered by traits and phylogeny at a higher elevation, with this effect marginally weaker for functional (*R* ^2^ = .43) than phylogenetic (*R* ^2^ = .47) composition (Figure 2). This weaker functional trend reflected some tendency for overdispersion among functional traits at middle elevations as well as lower elevations (see Figure 2b)—implying apparent niche partitioning among species in these locations.

### 3.4 Phylogenetic signal and traits

**FIGURE 3:**
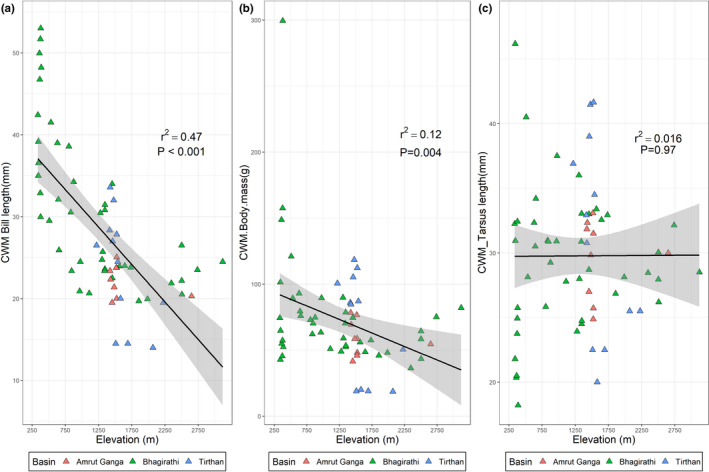
Trends of community weighted mean (CWM) values for the three functional traits (bill length (a), body mass (b), and tarsus length (c)) of river bird communities from different river basins plotted along the elevation gradient. The straight lines were fitted with a linear regression model and the *R* ^2^ values and *p* values are listed in each figure

In addition to the functional and phylogenetic clustering with increasing elevation, there was a significant phylogenetic signal in some of the functional traits of river birds as indicated by *K* and K* values (*p* < .05) for body mass (*K* = 1.2332), bill size (*K* = 1.4098), and tarsus length (*K* = 1.0725) (Table 1). In other words, similarities in these traits between species reflected strong phylogenetic effects. Among these three traits with a strong phylogenetic signal, CWM values for body mass and bill length declined with elevation, but there was no such effect in tarsus length (Figure 3). In contrast, K values for body size, tail length, and breeding traits indicated more substantial variation among related taxa, although only for body size was this effect formally significant (Table 1). Judged on Moran's I values, tarsus length and body mass had positive values while bill length had a negative autocorrelation with phylogenetic distance (Figure S5).

**TABLE 1:** Traits used to measure functional diversity and phylogenetic signals among breeding river birds in the Western Himalaya, India. The table gives Blomberg’s *K* values with significance values (in parentheses)

Trait	Blomberg's *K*	K*
Body mass (g)	1.23 (0.004)	1.13 (0.009)
Body size (mm)	0.68 (0.01)	0.63 (0.018)
Breeding months (number of months)	0.39 (0.172)	0.43 (0.167)
Clutch size (maximum number of eggs)	0.54 (0.027)	0.60 (0.023)
Bill length (mm) (from skull)	1.41 (0.001)	1.38 (0.001)
Tail length (mm)	0.28 (0.283)	0.31 (0.259)
Tarsus length (mm)	1.16 (0.003)	1.16 (0.008)

## 4 DISCUSSION

These data confirm the multifaceted changes in environmental conditions along Himalayan rivers over their large altitudinal range (Manel et al., 2000), in turn accompanied by pronounced variations in the community composition and trait character of river birds. Functional distances between co‐existing species decreased with increasing elevation after controlling for SR such that only a subset of traits persisted (Figure 2). Communities at higher altitudes shifted toward species with smaller bodies, shorter tarsi, smaller bills, and a greater tendency to feed as insectivores in the riparian zone or on aquatic prey. These patterns are consistent with the hypothesis that altitudinal trends affect these communities through environmental filtering (Dehling et al., 2014; Hanz et al., 2019; Vollstädt et al., 2017). They also echo similar filtering effects, for example, on ground beetles along a land disturbance gradient (Ribera et al., 2001), plants along a salinity gradient (Pavoine et al., 2011), ants along a complexity gradient (Weischer et al., 2012), bats across a gradient of forest fragmentation (Farneda et al., 2015), and birds with urbanization (Evans et al., 2018). Beyond these filtering effects, however, phylogenetic dispersion also declined with increasing altitude, illustrating for the first time that bird species composition along Himalayan rivers is constrained by phylogenetic origins: passerines, and specifically, muscicapids or their near relatives, dominated higher altitude rivers.

A range of caveats affect interpretation in studies like ours where survey data are used to test hypotheses. Above all, the evolutionary phenomena implied in our analyses occur over temporal and spatial scales that preclude straightforward experimentation. Large‐scale surveys of this type provide one of the few pragmatic methods of capturing large‐scale phenomena, but need appropriate design to eliminate potential confounds as well as data that corroborate the ecological or evolutionary processes inferred from correlations (Manel et al., 2000). In support of our approach, our design involved surveys that were replicated across regions, and observations that we took as robust representations of past evolutionary processes—such as phylogenetic relatedness or trait expression. Nevertheless, there are well‐known challenges in understanding how trait data or phylogenies reflect ecological processes (Cadotte et al., 2019). Furthermore, functional approaches fail to account for within‐species variations across populations while the phylogenetic approach can inflate signals related to certain traits (Zhao et al., 2020). At a more empirical level, some parts of our analysis would have been improved by more detailed data. Feeding traits, for example, were represented only crudely by categorizations of prey use, yet river birds can make precise selection for different prey types when foraging. This includes targeting prey of specific size, elemental composition, accessibility, and ease of handling (Ormerod & Tyler, 1991). Potentially, even more important in the context of niche use and limiting similarity is the extent to which riparian and river birds in the Himalayan mountains use subtly different components of the available prey base. This is apparent among the common insectivores whose diet appears to be partitioned along dimensions of prey size, taxonomic composition, and capture method (aerial vs. terrestrial vs. aquatic) (Buckton & Ormerod, 2008). However, incomplete dietary information from several of the species in our study precluded a more detailed dietary assessment. In a similar vein, measurements of the availability or abundance of prey used by any of the species were beyond the capabilities of this study, even though prey abundance is known to influence the density of river birds (Ormerod, 1985; Ormerod & Tyler, 1991). A further limitation is that both of our field surveys and data analysis focused on the breeding season, yet several of the species in our study are altitudinal migrants that descend to lower elevations in winter. As a consequence, our investigation is likely to have reflected evolutionary effects during the breeding period when resources demands and selection pressures are likely to be large (Verhulst & Nilsson, 2008).

Notwithstanding these caveats, our study revealed clear relationships among river character, species traits, and community composition of river birds in the Himalayan Mountains aided by the increasingly used RLQ analysis (Ribera et al., 2001). Here, over the largest altitudinal range on Earth, species composition and trait representation changed dramatically as several species of kingfishers, River Lapwing, Common Sandpiper, Blue Whistling Thrush, and Spotted Forktail gave way at a higher altitude to a generally smaller, insectivorous and functionally clustered array of species such as Plumbeous Water Redstart, White‐capped Water Redstart, Brown Dipper, and Little Forktail. The latter group of passerines that breed along high‐elevation river reaches come from several genera (Figure 1c) and are morphologically adapted for different foraging techniques such as fly catching, ground gleaning, and aquatic foraging in aquatic, bankside, and riparian habitats. Besides tracking the trends in habitat structure and vegetation pattern assessed here, these community changes also reflect well‐known altitudinal trends in temperature, nutrient status, oxygen concentrations, discharge patterns, and sediment regimes that have major effects on fish densities, invertebrate abundances, and other factors influencing prey availability (Ormerod et al., 1994). The resulting heterogeneity in habitat character and productivity in this region have given rise to the Earth's greatest diversity of birds adapted to high energy, montane river systems where their selective habitat use, foraging methods, and niche partitioning are consistent with resource segregation (Buckton & Ormerod, 2002, 2008). These established patterns add to the support from our data for the hypothesis that river habitat templates have influenced trait distributions within river bird communities through the evolutionary history of the species involved.

In addition to major altitudinal trends in community composition and trait expression among Himalayan river birds, we found that species were assembled non‐randomly along the elevation gradient into communities with distinct phylogenetic origins and functional character. Although reflecting patterns among a small group of species, the strength of this phylogenetic signal implies that historical contingency has influenced trait–environment relationships and river bird communities in the Himalaya, particularly at the highest altitudes. It is especially noteworthy that three of the four species most abundant at high altitudes were Muscicapidae—Plumbeous Water Redstart, White‐capped Water Redstart, and Little Forktail—an Old World family with large richness across the Himalayan region in general (Sinha, 2021). When communities of organisms are shaped predominantly by environmental conditions, their composition is typically aggregated by similar trait compositions in similar habitats, irrespective of evolutionary history or phylogenetic relatedness (Poff, 1997; Southwood, 1977). This contrasts with our case where a strong phylogenetic signal in composition and functional traits reflected circumstances where related species co‐occurred because of shared environmental requirements, and similar general morphology and behavioral character (Webb et al., 2002). Assuming that trait values (body mass, bill length, and tarsus length) reflected niche occupancy, the strong phylogenetic signals in our data suggest that functional traits and niche occupancy were constrained by phylogeny, at least at a higher elevation. Interestingly, however, some aspects of trait expression departed from the expectations of phylogenetic effects more than others: phylogeny was reflected in body mass, bill size, and tarsus length more than in body size, tail length, and breeding traits (Table 1). Moreover, at middle elevations, overdispersion among traits provided some evidence of niche partitioning among the most species‐rich communities (See Figure 2b). We suggest that community assembly in high‐altitude river birds must therefore reflect a blend of phylogenetic constraint and habitat filtering coupled with some proximate niche‐based selection of trait character for specialization (Morelli et al., 2019; Reif et al., 2016). This effect is particularly well illustrated in the forktails (*Enicurus* spp.), in which tail length in the Little Forktail is substantially reduced in comparison to its congeners and potentially linked to its highly specialized foraging niche around the splash zone of large boulders in highly turbulent flows (Buckton & Ormerod, 2008). Similarly, White‐capped Water Redstart and Plumbeous Water Redstart contrast in body size, with smaller size in the latter potentially facilitating energy efficiency in its extensive use of aerial foraging. Further detailed assessments of trait expression, function, and niche partitioning among Himalayan river birds are an interesting research avenue.

### 4.1 Broader implications: conservation and environmental change

As well as their relevance to evolutionary influences on river birds over the large altitudinal range of the Himalayan Mountains, our findings have broader implications for biodiversity conservation. Human impacts on rivers tend to simplify structural complexity, reduce connectivity, and impair water quality, and across the world, these processes are contributing to the decline or elimination of specialist organisms and population reductions that are among the fastest of any global ecosystem (Bower & Winemiller, 2019; Evans et al., 2018; Tickner et al., 2020). These effects arise because river catchment ecosystems are both hotspots for biological diversity and hotspots for resource exploitation (Strayer & Dudgeon, 2010). Both these factors have parallels with our data. First, at a global level, the association between overall bird richness and habitat heterogeneity is a well‐known phenomenon, especially for species that are specialized for particular habitat types—in this case, high‐energy rivers (Larsen et al., 2010; Robinson et al., 2002). Specialist river birds have developed unparalleled richness and niche specificity in the Himalaya reflecting both the complex relief and productivity in this region so that major habitat impairment could have effects of global significance (Buckton & Ormerod, 2002). Secondly, these same river environments face multiple pressures, for example, from climate change, catchment conversion to agriculture, pollution, hydropower, and water‐resource exploitation (Manel et al., 2000; Sinha et al., 2019). Some species in our study were associated with the least modified river reaches where bank vegetation, geomorphological structure, and flow patterns were unimpaired and expected to support abundant prey (Ormerod & Tyler, 1987, 1991; Sinha et al., 2019). Possible effects of habitat modification were also apparent in the different river basins surveyed, for example, where the river reaches in the Bhagirathi basin were modified for hydropower development (Figure 1a). If our interpretation is correct—that riparian and riverine habitat features act as environmental filters that structure river bird assemblages locally—it is likely that anthropogenic effects on rivers will modify these filtering processes and alter community composition unless checked by conservation action. Particular phylogenetic groups of species are at risk.

## 5 CONCLUSION

Overall, these data have both regional and general significance. Regionally, they provide explanations for changing community composition and trait expression in Himalayan rivers. More generally, they expand the understanding of how trait distributions and assemblages are the result of a complex interplay between trait filtering along environmental gradients coupled with evolutionary processes. There exists a clear phylogenetic imprint that contributes to contemporary species trait–habitat relations in river bird assemblages in the Himalayan Mountains. In the light of large‐scale human alterations to the biosphere, represented particularly strongly in rivers, models of trait–environment relationships like ours can be instrumental in predicting future range shifts in the distribution of species and traits. Our study reiterates that the simultaneous assessment of phylogenetic relatedness among co‐existing species with trait–habitat analyses can benefit the understanding of species assembly patterns across regional fauna.

## AUTHOR CONTRIBUTIONS

**Ankita Sinha:** Conceptualization (equal); data curation (equal); formal analysis (equal); funding acquisition (equal); investigation (equal); methodology (equal); project administration (equal); resources (lead); validation (lead); visualization (lead); writing – original draft (lead); writing – review and editing (lead). **Nilanjan Chatterjee:** Conceptualization (equal); formal analysis (equal); investigation (equal); methodology (equal); visualization (equal); writing – original draft (equal); writing – review and editing (equal). **Ramesh Krishnamurthy:** Funding acquisition (equal); project administration (equal); resources (equal); supervision (equal); writing – review and editing (equal). **Steve J. Ormerod:** Investigation (equal); methodology (equal); supervision (equal); validation (equal); writing – review and editing (equal).

## CONFLICT OF INTEREST

The authors have no conflict of interest to declare.

6

## Supporting information


Appendix S1

## Data Availability

The data that support the findings of this study can be found in Figshare. https://doi.org/10.6084/m9.figshare.19796227.v1
