# Supplementary material for: Community assembly, functional traits, and phylogeny in Himalayan river birds
Source: Ecol Evol. 2022 Jun 17;12(6):e9012. doi: 10.1002/ece3.9012 (PMC9204853; doi:10.1002/ece3.9012)
Supplement: Supplementary file 1 — Appendix S1 [file ECE3-12-e9012-s001.docx]

**Supplementary material**

**Community assembly, functional traits and phylogeny in Himalayan river birds**

Ankita Sinha, Nilanjan Chatterjee, Ramesh Krishnamurthy, Steve J. Ormerod


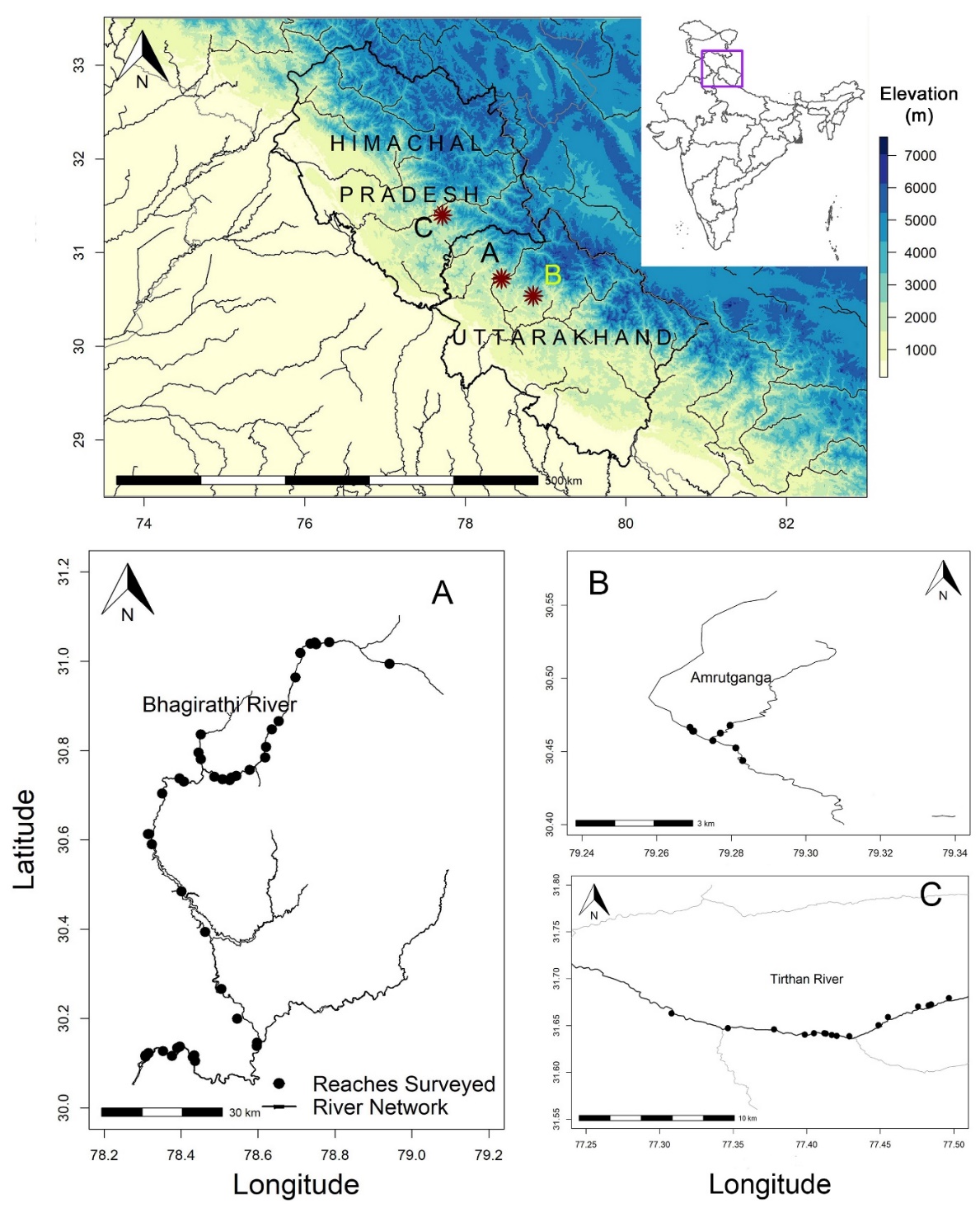


FIGURE S1. Map showing the locations of (a) the Bhagirathi and Amrut Ganga river basins in Uttarakhand and (b) the Tirthan river basin in Himachal Pradesh in India.


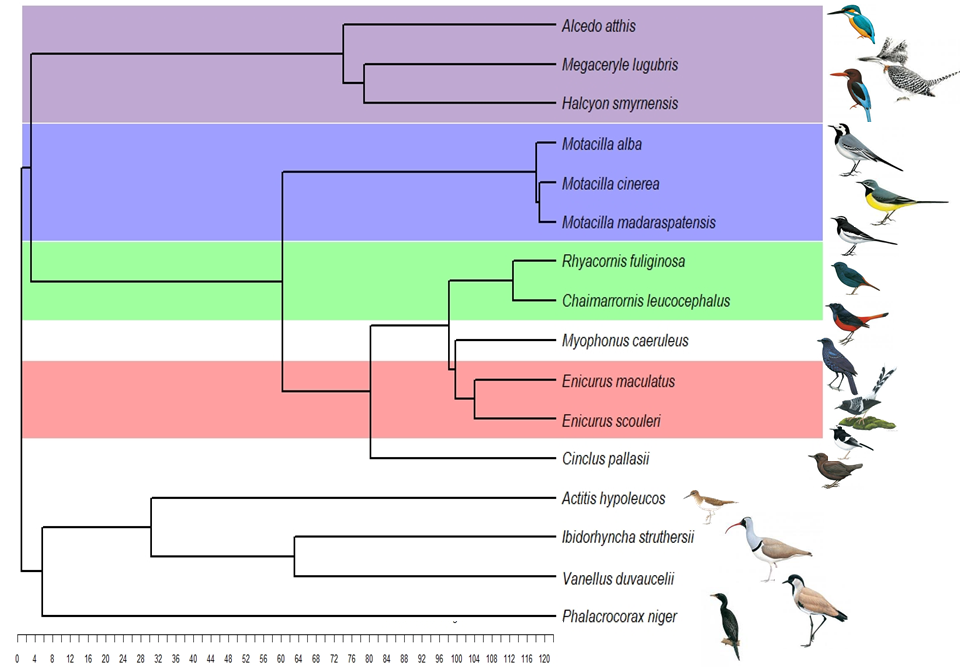


FIGURE S2. Dendogram showing the phylogeny of rivers birds in the Bhagirathi, Amrut Ganga and Tirthan river basins in Western Himalaya, India. Phylogenetic tree for the species was prepared by trimming from the original phylogeny available from [www.birdtree.org](http://www.birdtree.org) (Jetz et al. 2012).


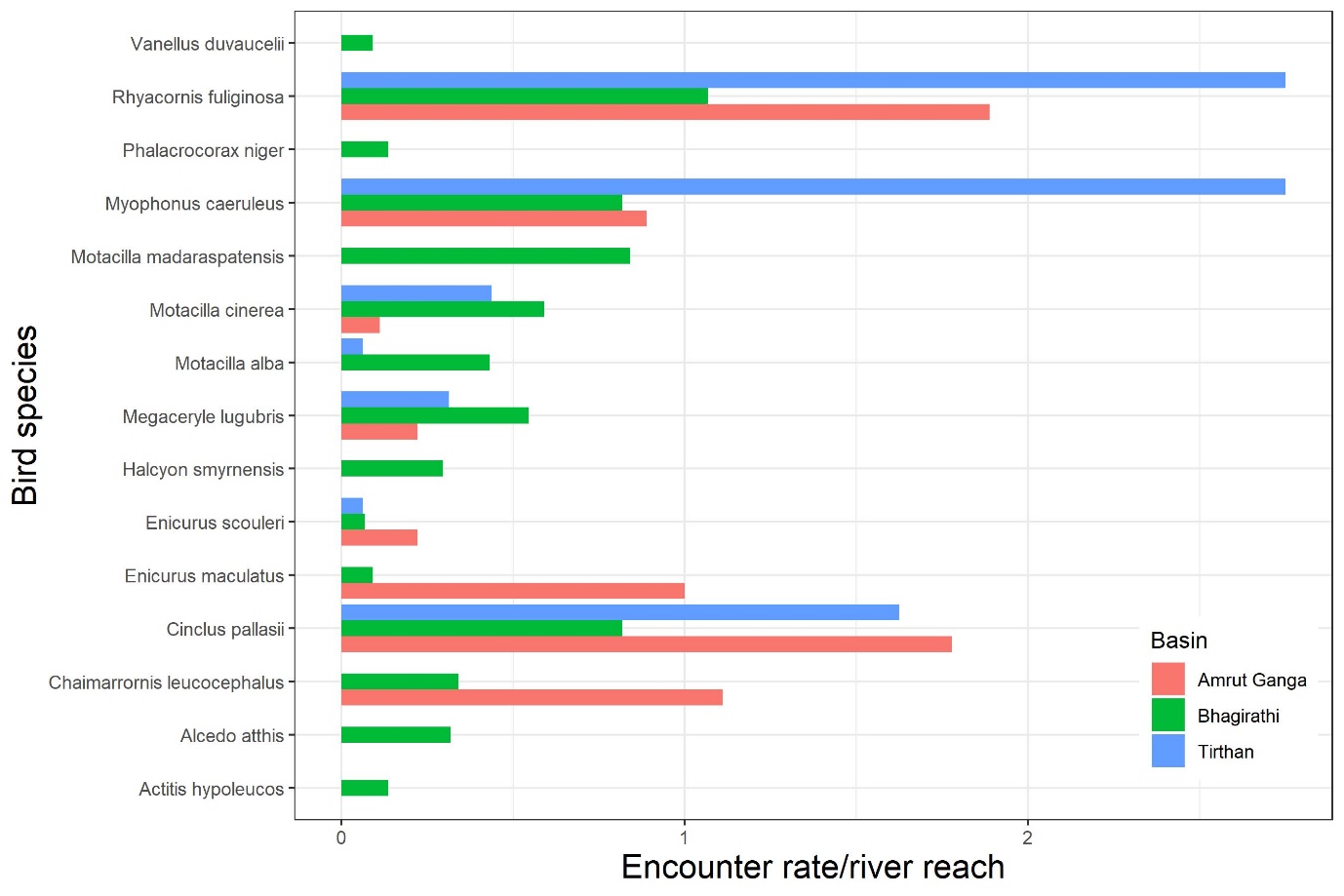


FIGURE S3. Encounter rate (number of birds recorded per river reach) of different river bird species in the Bhagirathi (N =15), Amrut Ganga (N =8) and Tirthan (N=8) rivers. A total of forty-three, nine and sixteen river reaches were sampled in the Bhagirathi, Amrut Ganga and Tirthan river basins respectively.


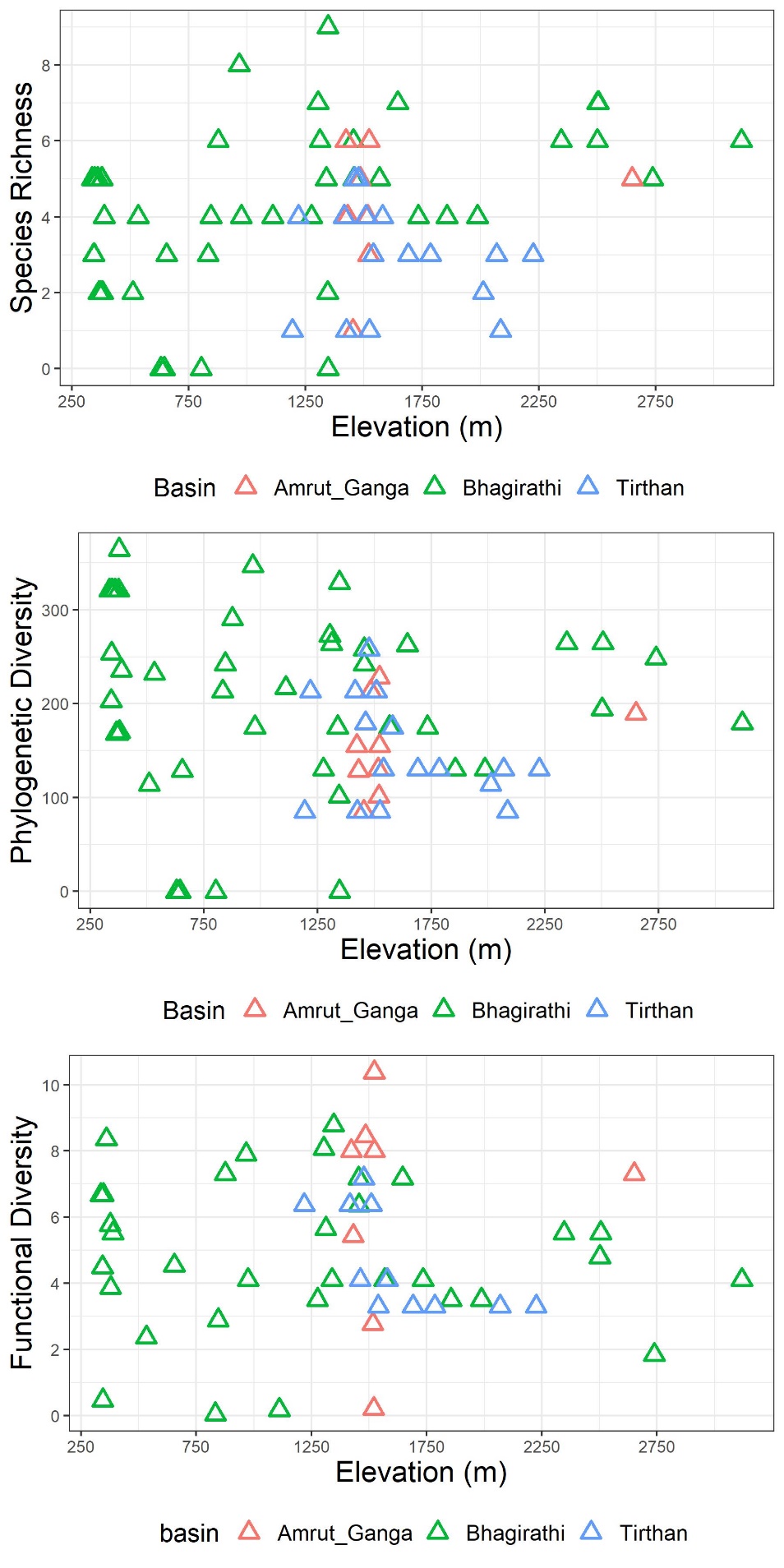


FIGURE S4. Plots showing elevational patterns of species richness, phylogenetic diversity (Faith’s PD) and functional diversity (FRic) of breeding river birds in the western Himalaya.


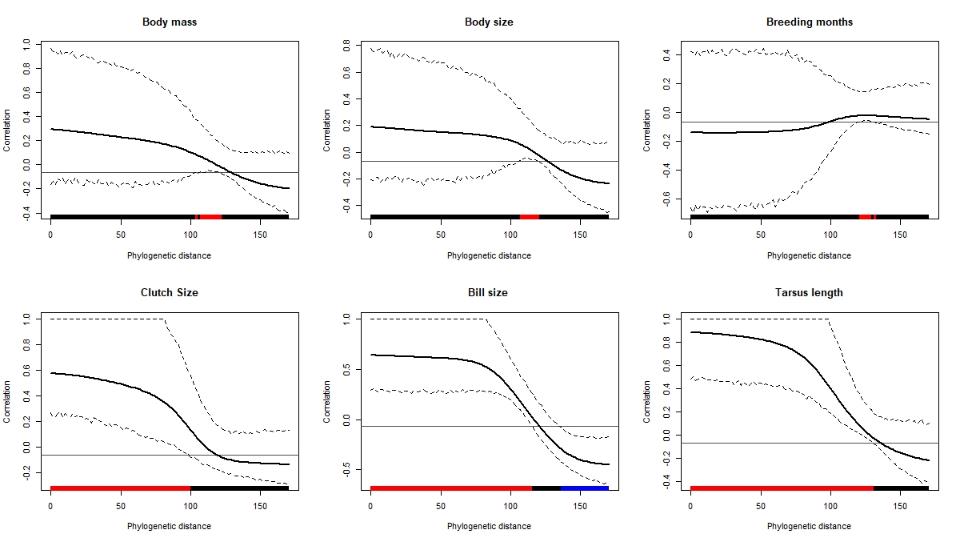


FIGURE S5. Phylogenetic correlogram for the six traits (diet, foraging behaviour, foraging substrate, and habitat) for 15 bird species recorded in field surveys. The figure shows the mean phylogenetic signal (solid bold black line represents the Moran's I index of autocorrelation) with a 95% confidence interval resulting from 100 bootstraps (dashed black lines represent both lower and upper bounds of the confidence interval). The coloured horizontal bars show whether the autocorrelation is significant: red is a significant positive autocorrelation, blue is a significant negative autocorrelation and black is a non-significant autocorrelation.

TABLE 1. Summary of the RLQ analysis of breeding communities of river birds in the Western Himalaya showing the first three ordination axes from the species abundance (L), trait (Q) and habitat (R) matrices. Trends in habitat character, traits and species along each axis are shown in Figure 2

| Total inertia= 1.34 | | | |
| --- | --- | --- | --- |
| Projected inertia (%) | | | |
|  | Ax1 | Ax2 | Ax3 |
|  | 80.633 | 9.675 | 7.05 |
| Eigenvalue decomposition | | | |
|  | Eig | Covariance | Correlation |
| eig1 | 1.080 | 1.039 | 0.389 |
| eig2 | 0.129 | 0.360 | 0.264 |
| Inertia and co-inertia R: | | | |
|  | Inertia | Max | Ratio |
| eig1 | 2.464 | 2.982 | 0.826 |
| eig1+2 | 3.844 | 5.317 | 0.723 |
| Inertia and co-inertia Q: | | | |
|  | Inertia | Max | Ratio |
| eig1 | 2.894 | 3.376 | 0.857 |
| eig1+2 | 4.238 | 6.408 | 0.661 |
| Correlation L: |  |  |  |
|  | Correlation | Max | Ratio |
| eig1 | 0.389 | 0.860 | 0.452 |
| eig2 | 0.264 | 0.572 | 0.461 |
